# Exploring Teachers' Knowledge and Attitudes Toward Attention Deficit Hyperactivity Disorder and Its Treatment in a District of Turkey

**DOI:** 10.7759/cureus.45342

**Published:** 2023-09-16

**Authors:** Berhan Akdağ

**Affiliations:** 1 Child and Adolescent Psychiatry, Silifke State Hospital, Mersin, TUR

**Keywords:** teacher, perception, knowledge, attention deficit hyperactivity disorder, adhd

## Abstract

Background

Teachers are pivotal in integrating children with attention deficit hyperactivity disorder (ADHD) into academic and social contexts. Their comprehension of and attitudes toward ADHD significantly influence the inclusion of these children. This study was conducted to assess teachers' knowledge, attitudes, and perceptions about ADHD and its treatment within a representative sample from Turkey.

Methods

An online self-administered questionnaire was formulated to gauge teachers’ knowledge, attitudes, and perceptions related to ADHD and its treatment.

Results

Of the respondents, 57.7% accurately identified that ADHD is more commonly present in boys. Furthermore, a majority of teachers (60.8%) correctly answered the question related to the comorbidity of ADHD and learning disabilities. However, 20.3% of teachers believed that ADHD medications were addictive, with 9.7% expressing reluctance to use such treatment for their children if needed.

Conclusion

The results highlight the need for revising the current training curricula for novice teachers and providing additional training for experienced teachers. Such initiatives should aim to rectify any negative perceptions and attitudes toward ADHD held by teachers.

## Introduction

Attention deficit hyperactivity disorder (ADHD) is a prevalent neurodevelopmental disorder typically emerging during childhood. Epidemiological data suggest it affects an estimated 5% to 11% of individuals aged 18 years and under, with a higher prevalence observed among males compared to females [1]. Individuals with ADHD commonly experience difficulties maintaining attention (e.g., easy distraction and forgetfulness in daily activities) and regulating impulsive behavior (acting without considering potential consequences), and they may exhibit signs of hyperactivity (e.g., running in inappropriate situations and excessively talking) [2]. Additionally, they often face adversities in academic and social contexts attributable to abbreviated attention spans, uncontrolled impulsivity, and emotional dysregulation [3].

Children with ADHD in classroom environments

Classroom settings present a unique set of challenges for children with ADHD, as they necessitate behaviors that inherently oppose ADHD's key symptoms. Prior research has revealed that ADHD children frequently encounter numerous school-related difficulties attributable to hyperactivity, inattention, and impulsivity, which are hallmark symptoms of the disorder. Interestingly, gender-specific variations in symptom manifestation may be observed. For example, boys predominantly exhibit externalized symptoms such as hyperactivity and impulsivity, whereas girls tend to display more internalized symptoms, including inattention, diminished self-esteem, and depression [4].

Academic performance of children with ADHD often suffers, resulting in grade retention or even school suspension. Their unique symptom profiles frequently lead to diverse problematic behaviors in the school context. Children with predominantly impulsive symptoms may, for instance, disrupt class by interjecting without permission or engaging other students at inappropriate times. Conversely, those with primarily inattentive symptoms might struggle to adhere to instructions and rules, maintain focus on tasks, or accomplish assigned work. Hyperactive children typically find it challenging to remain seated, avoid playing with unrelated objects during tasks (e.g., fiddling with a pencil during silent reading), cease chair-rocking, or halt repetitive hand or foot tapping [5]. However, it is important to note that most children with ADHD experience difficulties related to at least two of these core symptom groups. In addition, for an ADHD diagnosis, the symptoms must be present in at least two settings, such as at home, school, or with friends [6].

Given ADHD's symptomatic nature, it is expected that associated behaviors would impact peer relationships in school environments. Children with ADHD may exhibit controlling, disruptive, and aggressive behaviors, likely leading to negative perceptions among their peers. Consequently, these children may find themselves ostracized from peer-group play activities. Furthermore, children with ADHD might struggle to accurately interpret social cues, thereby responding inappropriately. A study by Gresham et al. [7] revealed that approximately 70% of children with ADHD have unreciprocated friendships, and typically developing children often express reluctance to befriend their ADHD-affected peers, especially those demonstrating hyperactivity. In summary, ADHD can significantly impact children's social functioning and contribute to numerous negative mental health outcomes, including low self-esteem, depression, and social anxiety.

ADHD and teachers

Educating students diagnosed with ADHD can present significant challenges for teachers. These educators often encounter difficulties in their professional capacities, such as managing the unique behavioral, attentional, and learning needs of these students. Manifestations of ADHD such as constant fidgeting, abruptly leaving their seat, interrupting discussions, and causing class-wide disruptions can lead to feelings of frustration for the educators [8]. The characteristics inherent to ADHD often instigate a sense of pessimism in teachers, alongside feelings of guilt and inadequacy stemming from perceived shortcomings in reaching and adequately addressing the learning needs of these students.

The role of educators in relation to ADHD is multifaceted and of considerable importance. Initially, teachers often play a crucial role in the early identification of ADHD. The diagnosis process of this condition does not rely on a single test; instead, it depends on an extensive review of the child's history and behavioral observations across multiple contexts, which frequently include ADHD symptom checklists rated by parents and teachers [9]. Given their unique position, teachers often become the first to recognize or suspect ADHD in students as the symptoms typically interfere with academic performance or disrupt classroom cohesion. This early recognition is vitally important, as untreated ADHD is associated with a range of negative life outcomes such as accidental injuries, criminal behavior and delinquency, educational underperformance, precocious and increased substance use, earlier engagement in sexual activities, and associated risks, including adolescent pregnancy [10]. Furthermore, teachers have the capacity to help students with ADHD achieve their full academic potential. This can be accomplished through the implementation of evidence-based classroom interventions and the establishment of a supportive learning environment. In addition, teachers have the opportunity to positively influence the social functioning of students with ADHD. This can be achieved by highlighting their strengths, offering positive reinforcements, and cultivating an environment conducive to the demonstration of their skills. Importantly, teachers' attitudes and behaviors toward students with ADHD can significantly influence the perceptions of their peers.

Teachers’ perceptions, attitudes, and knowledge concerning ADHD

Teachers play a pivotal role in both the identification and management of ADHD. However, their effectiveness in these roles can be significantly influenced by a range of factors, including their understanding and perception of ADHD. Teachers who lack accurate knowledge or harbor misconceptions about ADHD may fail to recognize or utilize evidence-based interventions and accommodations designed to support students with ADHD in the classroom. In contrast, teachers who possess a more comprehensive understanding and display a positive attitude toward ADHD are generally more inclined to employ effective strategies. These strategies not only aid children with ADHD in their academic achievement but also alleviate their symptoms. Previous studies have underscored a significant correlation between a teacher's knowledge of ADHD and their confidence in effectively teaching children with ADHD, fostering an inclusive classroom environment, and managing disruptive behaviors [11,12]. However, societal stigma, which is not confined to teachers, can negatively impact students' attitudes toward education. This can potentially result in their disengagement from learning and eventual discontinuation of schooling.

Teachers' attitudes toward ADHD may also diverge, ranging from positive to negative, and are likely to be shaped by their perceived teaching efficacy and expectations. Additionally, attitudes can differ depending on various factors such as the age, gender, severity of symptoms, and academic performance of the children with ADHD. Interestingly, classroom exposure to ADHD may contribute to teachers' knowledge of the disorder. Sciutto et al. [13] reported a positive correlation between prior exposure to ADHD and the depth of ADHD knowledge among teachers. Yet, Kos et al. [14] found no evidence to support this association.

The present study

In the context of academic and social inclusion for children with ADHD, the role of teachers proves to be vital. Their knowledge and attitudes toward ADHD significantly contribute to fostering an inclusive environment for these children. Globally, substantial research has been conducted to understand teachers' knowledge and attitudes toward students with ADHD. However, there is a knowledge gap in the context of Turkish teachers that has been attempted to be filled by a limited number of studies [15]. This gap is particularly concerning given that a recent nationwide study in Turkey reported a higher prevalence of ADHD among children in the second to fourth grades (19.5% without impairment and 12.4% with impairment) than the global average [16]. Moreover, children with ADHD in Turkey may miss out on the free support and educational resources available to them due to the underdiagnosis of ADHD. Therefore, the current study sought to evaluate the knowledge, attitudes, and perceptions concerning ADHD and its treatment among a district sample of teachers in Turkey.

A version of this paper was presented at the 32nd National Congress of Child and Adolescent Psychiatry, Istanbul, Türkiye, 2023.

## Materials and methods

Participants and procedure

An online self-administered questionnaire, modeled after prior surveys exploring teachers’ knowledge, attitudes, and perceptions about ADHD [17], was developed. The questionnaire addressed areas pertinent to clinical practice and potential impediments to diagnostic or therapeutic processes.

Initially, participants were asked, “What do you believe is the prevalence of ADHD?” to gauge their perceptions about the prevalence of ADHD. This was followed by assessing their general knowledge about ADHD through responses to five statements. An option for “I have no idea” was incorporated to diminish the chances of guesswork, thereby enhancing the probability that the responses truly reflected the respondents’ knowledge [17]. The third question posed was, “Are the medications used to treat ADHD addictive?” Finally, the teachers’ attitudes and perceptions concerning ADHD and its treatment were ascertained via a 3-point Likert scale response to four items (1 = yes, 2 = unsure, 3 = no).

Approval for the study was granted by the Provincial Education Directorate. The online survey was distributed among schools in Silifke, a district situated along the Mediterranean coast in Turkey, during a brief sampling period (December 23 to December 30, 2022). Silifke boasts 117 educational institutions (including preschools, elementary schools, secondary schools, and high schools), employing approximately 2,300 teachers during the study period. Prior to initiating the survey, an online consent form detailing the data collection procedure and study objectives was provided. Assurance was given that participation was anonymous and voluntary. After perusing the information sheet and giving consent, willing participants gained access to the survey. Ethical approval was granted by the Toros University Scientific Research and Publication Ethics Committee (Approval no: 2022/160), with the study adhering to the principles outlined in the Helsinki Declaration.

Statistical analysis

The data were analyzed utilizing SPSS Version 28.0 (IBM Corp., Armonk, NY). Descriptive statistics are presented as means (standard deviation) and percentages (%). The findings were subsequently depicted in tables and graphs.

## Results

The online survey was completed by 227 teachers, approximately 10% of the educators employed in Silifke. The mean age of the teachers was 42.30 years (SD = 7.90), with 17.2% possessing a tenure of 10 years or less. Of the total, 23.3% (n = 53) had undergone ADHD-specific training, and 59.5% had taught students diagnosed with ADHD. Detailed demographic information is provided in Table 1.

**Table 1:** Demographic characteristics of the participants (n = 227) ADHD, attention deficit hyperactivity disorder

Characteristics	
Age (years), mean (SD)	42.30 (7.90)
Gender, n (%)	
Female	147 (64.8)
Male	80 (35.2)
Branch, n (%)	
Pre-school teacher	31 (13.7)
Primary school teacher	94 (41.4)
Subject teacher	102 (44.9)
Work experience, n (%)	
10 years or below	39 (17.2)
11–15 years	49 (21.6)
16–20 years	57 (25.1)
21 years or above	82 (36.1)
Have you ever taught a child with ADHD? n (%)	
Yes	135 (59.5)
No	92 (40.5)
Have you ever participated in education on ADHD? n (%)	
Yes	53 (23.3)
No	174 (76.7)
Have you ever researched ADHD individually? n (%)	
Yes	132 (58.1)
No	95 (41.9)
Have you ever referred a child with suspected ADHD? n (%)	
Yes	110 (48.5)
No	117 (51.5)

**Figure 1:**
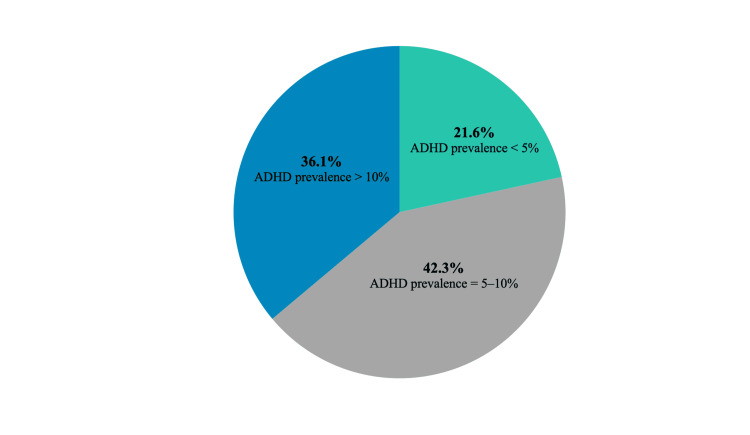
Teachers’ perceptions regarding the prevalence of ADHD ADHD, attention deficit hyperactivity disorder

Upon being queried about the prevalence of ADHD, 42.3% of teachers estimated it to fall between 5% and 10% (Figure 1).

The majority (57.7%) accurately identified a higher prevalence of ADHD in boys. Moreover, 60.8% correctly recognized the comorbidity of ADHD and learning disabilities. Conversely, a minor proportion believed ADHD was not a consequence of parental attitudes (18.5%) or that inattention and hyperactivity/impulsivity did not necessarily co-occur (41.4%). Table 2 summarizes teachers' responses to general knowledge-based questions about ADHD.

**Table 2:** Percentages of responses to questions related to general knowledge about ADHD *Correct responses ADHD, attention deficit hyperactivity disorder; LDs, learning disabilities

Questions	Response rate
Q1: What is true about the prevalence of ADHD in boys and girls?	
ADHD presents more frequently in boys*	57.7%
ADHD presents more frequently in girls	2.6%
ADHD is equally common in boys and girls	11.0%
I have no idea	28.6%
Q2: Inattention and hyperactivity/impulsivity must be present together.	
Yes	17.6%
No*	41.4%
I have no idea	41.0%
Q3: ADHD is a result of parental attitudes.	
Yes	54.6%
No*	18.5%
I have no idea	26.9%
Q4: LDs are more common in children with ADHD than others.	
Yes*	60.8%
No	8.8%
LDs are equally common in children with ADHD and others.	3.5%
I have no idea	26.9%
Q5: What is the primary treatment for ADHD?	
Medications/family education/behavioral therapy/counselling*	70.9%
Special education	27.3%
Others	1.8%

**Figure 2:**
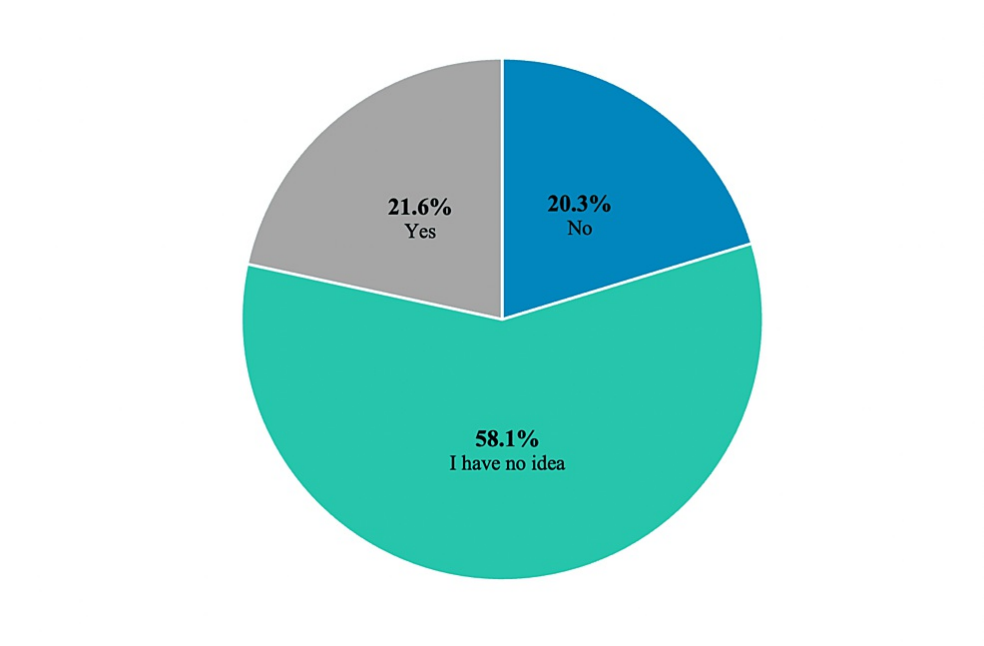
Teachers’ responses to the question: “Are medications used to treat ADHD addictive?” ADHD, attention deficit hyperactivity disorder

In response to the question “Are medications used for treating ADHD addictive?” 20.3% of respondents confirmed, whereas 58.1% expressed uncertainty (Figure 2).

**Figure 3:**
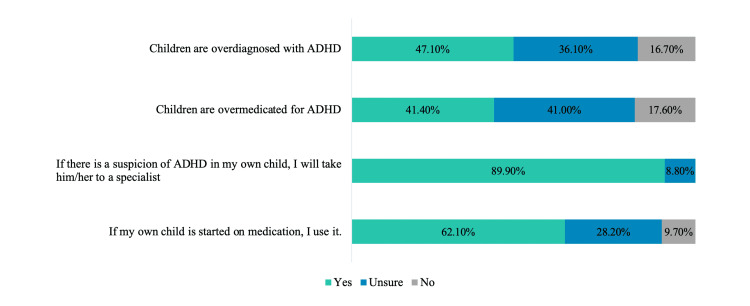
Teachers’ attitudes and perceptions toward ADHD and its treatment The percentages (%) of teachers' answers to the statements are given. ADHD, attention deficit hyperactivity disorder

Attitudinal responses toward ADHD and its treatment are delineated in Figure 3. Approximately half (47.1%) of the teachers believed that ADHD has been overdiagnosed in children, and a small percentage (9.7%) indicated that they would refuse treatment for their children if an ADHD diagnosis was made.

## Discussion

In this study, we observed that 20.3% of the participating teachers believed that medications used to treat ADHD were potentially addictive, and 58.1% remained uncertain. Notably, 9.7% of participants expressed that they would refrain from administering such treatments to their own children, suggesting a need for further clarity on the subject. ADHD and substance use disorders (SUDs) are known to coexist. Prior research indicates that children with ADHD are more prone to initiate alcohol abuse than their counterparts, and individuals diagnosed with ADHD are likely to engage in the use of alcohol and other substances at an earlier age [18]. Several factors, including inhibitory control deficits and behavioral problems, contribute to the relationship between ADHD and SUDs [19]. Thus, the risk of addiction is inherently tied to ADHD rather than its pharmacological treatment. Additionally, early intervention with pharmacotherapy for ADHD can mitigate the risk of developing alcohol and other SUDs [20]. However, it is important to underscore the potential complications associated with ADHD pharmacotherapy when misused. Indeed, ADHD medications have the potential to be addictive, particularly if they are misused. Moreover, the global prevalence of stimulant misuse is on the rise due to increased prescription [20]. Clinicians, therefore, should pay particular attention to individuals with SUDs and college students who, despite not being diagnosed with ADHD, falsely believe that these medications can enhance concentration and academic performance [21].

According to only 18.5% of participants, the assertion that “ADHD is a result of parental attitudes” is misguided. The precise causes and risk factors for ADHD are not yet fully understood, though recent studies have underscored the significant influence of genetic factors in the development of ADHD [22]. Moreover, impaired neurotransmitter functioning, namely in norepinephrine and dopamine, is considered an underlying mechanism of ADHD. Consequently, certain medications are designed to alleviate ADHD symptoms by facilitating the release and function of dopamine and norepinephrine in the brain [23]. Nevertheless, misconceptions regarding the origins of ADHD persist in the general population, such as the unfounded notions that excessive beverage consumption, parental attitudes, or family chaos can cause ADHD. While these elements may exacerbate symptoms in children with ADHD [24,25], current evidence does not support the assertion that they are primary causative factors for the disorder [26].

The present study revealed that a significant majority (60.8%) of teachers recognized learning disabilities more frequently in children with ADHD compared to their peers. However, diagnosing ADHD remains a complex task due to the existence of multiple conditions that share symptomology with ADHD. For example, academic underachievement, a typical outcome for children with ADHD, is also observed in children with other disorders such as learning disabilities and intellectual disabilities. Interestingly, prior research indicates that 30-50% of children with ADHD concurrently exhibit a specific learning disability [27]. This correlation is particularly noteworthy as learning disabilities can obscure ADHD symptoms. Specifically, heightened attention demands brought about by learning difficulties are exacerbated when attention is compromised [28]. Given the distinct intervention strategies required, it is imperative that teachers possess adequate knowledge and awareness of both ADHD and learning disabilities to accurately differentiate these conditions.

Early childhood is when many children begin to show signs of ADHD, but a subset remains undiagnosed until adulthood. It is essential to recognize that ADHD can manifest in several forms: predominantly inattentive, predominantly hyperactive/impulsive, and the combined type [29]. Boys with ADHD often exhibit symptoms of hyperactivity, such as running and impulsivity, whereas girls generally display internalized symptoms, including inattentiveness, low self-esteem, and depression [4]. Girls with ADHD may be overlooked, as their symptoms are often less conspicuous and they demonstrate fewer behavioral problems. In the present study, 41.4% of participating teachers believed that inattention and hyperactivity/impulsivity need not coexist, and 57.7% stated that ADHD is more common in boys. However, 47.1% of the teachers felt that children are overdiagnosed with ADHD. Prior research has indicated that, despite having adequate knowledge of sex differences in ADHD, teachers often lack understanding about the disorder’s prevalence [30]. Studies examining teachers’ comprehension of ADHD prevalence are scarce, making it challenging to precisely determine their knowledge levels. It may be beneficial for teachers to better comprehend ADHD prevalence, thereby enabling them to accurately estimate the number of affected students in their classrooms. Furthermore, teachers unaware of actual prevalence rates might be more susceptible to over-identifying students with ADHD and avoiding referring them for diagnostic assessment [30]. Lastly, it is important to consider cultural diversities when addressing ADHD. In certain cultures, what is considered a symptom of ADHD in other cultures may not be attributable to ADHD. This may lead to a lack of referral for diagnostic evaluation. In this sample, some teachers (47.1%) believe that ADHD is overdiagnosed, and some (9.7%) are even defensive about their own children receiving possible treatment for ADHD. These perspectives could stem from cultural diversities.

This study sought to elucidate the understanding, attitudes, and perceptions of ADHD and its treatment among a cohort of teachers, marking the first such effort focusing on educators in Turkey’s rural regions. Nevertheless, the study’s findings and interpretations must be viewed within the context of its constraints. First, this study did not employ a validated questionnaire to assess teachers’ knowledge of ADHD. Instead, a custom five-item questionnaire was curated, zeroing in on aspects pertinent to clinical practice and those potentially disruptive to the diagnostic or therapeutic process. To enhance interpretability, descriptive analyses were undertaken at the item level, allowing for individual discussion of each item. Secondly, the generalizability of the findings may be somewhat limited. The study involved teachers in rural areas; the sample size and response rate were modest. Thirdly, the cross-sectional nature of the study prevented the tracking of changes in teachers’ knowledge and perceptions of ADHD over time. Lastly, the study did not probe the timing of ADHD-related training reported by some teachers, making it impossible to assess the continuity of their knowledge on the subject.

## Conclusions

This study's findings offer significant insights into teachers’ understanding, attitudes, and perceptions of ADHD. It was observed that teachers exhibit gaps in their ADHD knowledge in certain areas, underscoring the necessity for expanded training. This research also implies that it is crucial to revisit the existing curriculum of teacher training for newcomers and ongoing professional development programs for experienced educators. These programs should aim not only to broaden their knowledge but also to alleviate their negative perceptions and attitudes toward ADHD. Importantly, fostering teachers' comprehension and acceptance of the nature of ADHD can enhance learning efficacy for students with ADHD, potentially boosting their school attendance and educational engagement. While the importance of training programs is undeniable, their repeated reinforcement at regular intervals is of paramount importance.
